# Obliterating Bronchiolitis: Result of Iron Pill Aspiration

**DOI:** 10.7759/cureus.2571

**Published:** 2018-05-02

**Authors:** Andrew Chu, Alvin Krishna, Manju P Paul, James F Sexton, Kanish Mirchia

**Affiliations:** 1 Pulmonary and Critical Care Medicine, SUNY Upstate Medical University; 2 Internal medicine, SUNY Upstate Medical University; 3 Pathology, SUNY Upstate Medical University

**Keywords:** foreign body aspiration, bronchiolitis obliterans, iron pill aspiration

## Abstract

Foreign body aspiration occurs in all age groups, especially in children and the elderly. The aspiration of an organic foreign body such as iron sulfate can cause significant bronchial destruction via oxidizing necrosis. When iron comes into contact with bronchial mucosa, it gets oxidized from ferrous ions into a ferric form which is highly toxic to the mucosa causing severe inflammation, mucosal damage, and fibrosis. Physicians should be very prudent with prescribing iron sulfate or any other pills in individuals who are at high risk of aspiration. Diagnosis is based on the history of iron aspiration, intense airway inflammation or necrosis on bronchoscopic examination, and iron particles observed on pathology. Prompt diagnosis and management should take place to prevent further morbidities.

We report a case of 61-year-old female who was admitted to the hospital with a four-week history of aspirating iron pill. Computed tomography (CT) of the thorax showed ground glass infiltrates in the right lower lobe. She underwent flexible bronchoscopy which showed distal right bronchus intermedius (RBI) necrosis and stenosis with near-complete obstruction of distal RBI. She underwent multiple advanced bronchoscopic interventions with minimal improvement of the obliterated bronchus.

## Introduction

Iron pill aspiration syndrome (IPAS) is rarely reported in the literature although its consequences can be severe and life threatening. When iron comes into contact with the bronchial wall, it can cause inflammation, mucosal damage, and stenosis via forming granulomas or fibrosis [1]. The pathogenesis of this process is based on hydroxyl radial formation and lipid peroxidation of ferrous ions into the ferric form when iron comes into contact with the bronchial mucosa [2]. The hallmark of IPAS is characterized by a history of the patient aspirating the pill, intense airway inflammation, and iron particles seen on a bronchial biopsy [1].

We report a case of a 61-year old woman who presented with worsening cough and phlegm production four weeks after aspirating on an iron pill. The final diagnosis was made after a rigid bronchoscopy and tissue pathology. The case exhibits remarkable near obliterating bronchiolitis refractory to multiple advanced bronchoscopic therapies.

## Case presentation

**Figure 1:**
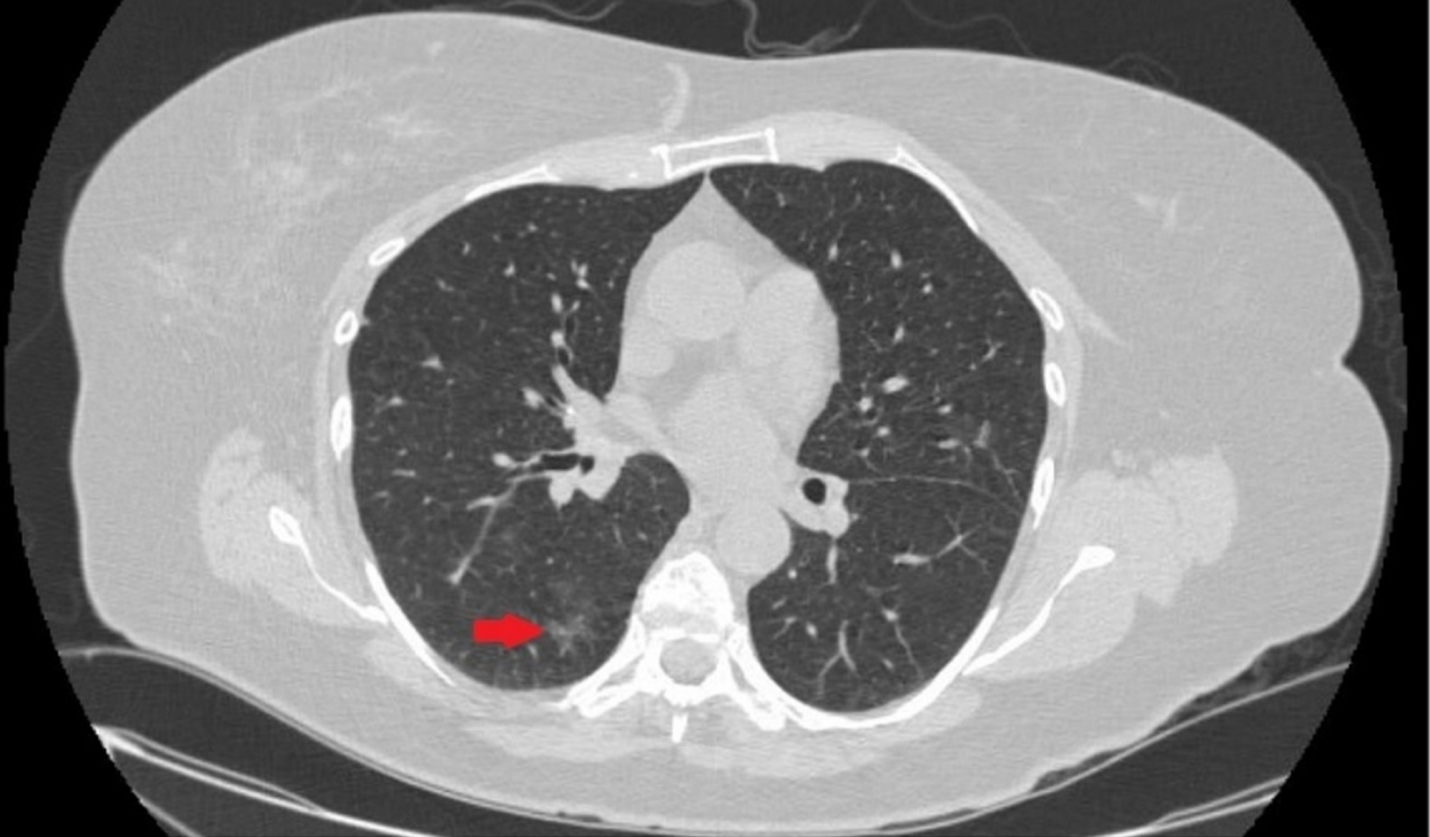
Computed tomography (CT) scan of the thorax showing ground glass infiltrate in the right lower lobe (red arrow)

A 61-year-old female was admitted to our hospital with a history of worsening cough and phlegm production for four weeks after choking on an iron pill. She had a history of hypertension, anemia, and immunoglobulin deficiency. The chest X-ray was unremarkable. Computed tomography (CT) of the thorax showed ground glass infiltrates in the right lower lobe (Figure 1).

**Figure 2:**
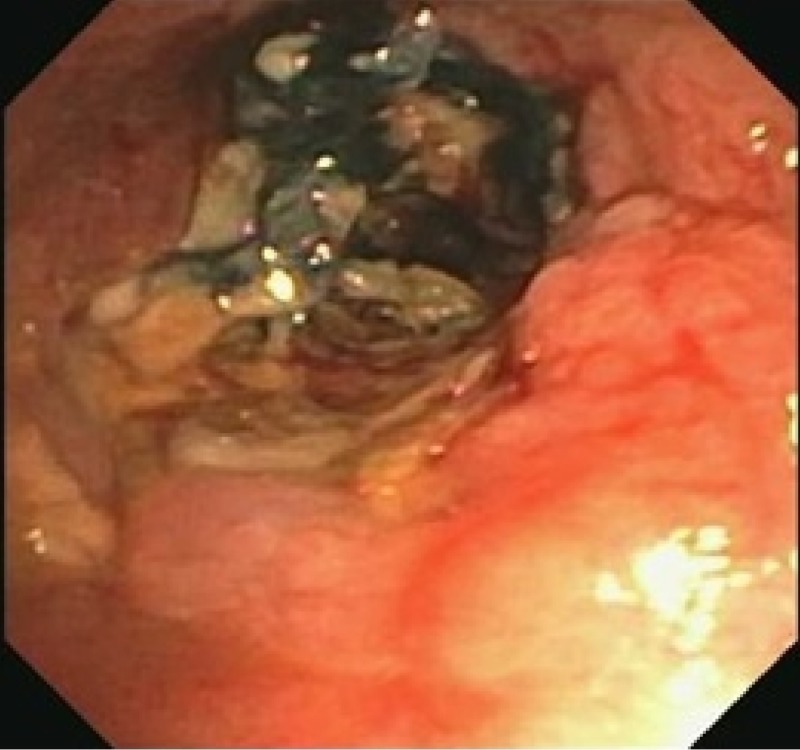
Bronchoscopic image of the bronchus intermedius showing extensive necrosis and obliteration

She underwent flexible bronchoscopy which showed distal right bronchus intermedius (RBI) necrosis and stenosis with near-complete obstruction of distal RBI (Figure 2).

**Figure 3:**
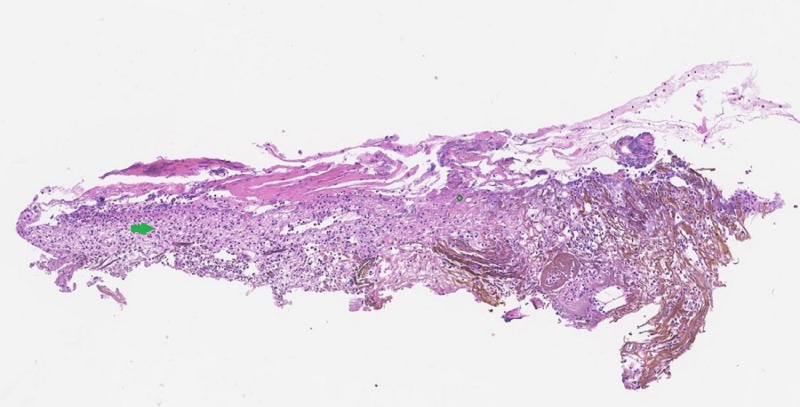
Haemotoxylin and eosin stain of the bronchial biopsy showing epithelial erosion and necrosis (green arrow)

**Figure 4:**
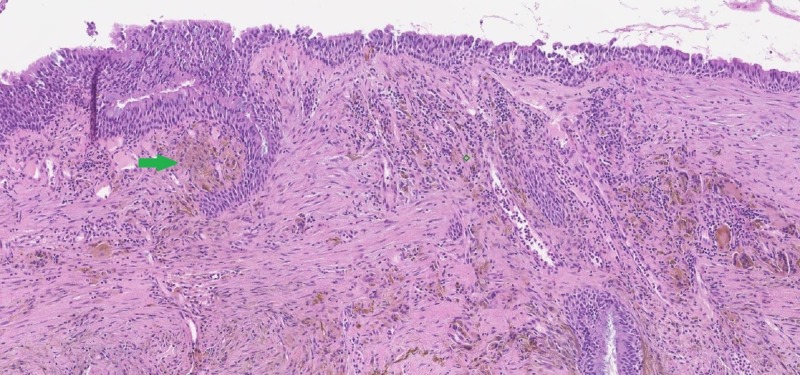
Haemotoxylin and eosin stain of the bronchial biopsy showing presence of unstained iron deposition (green arrow)

**Figure 5:**
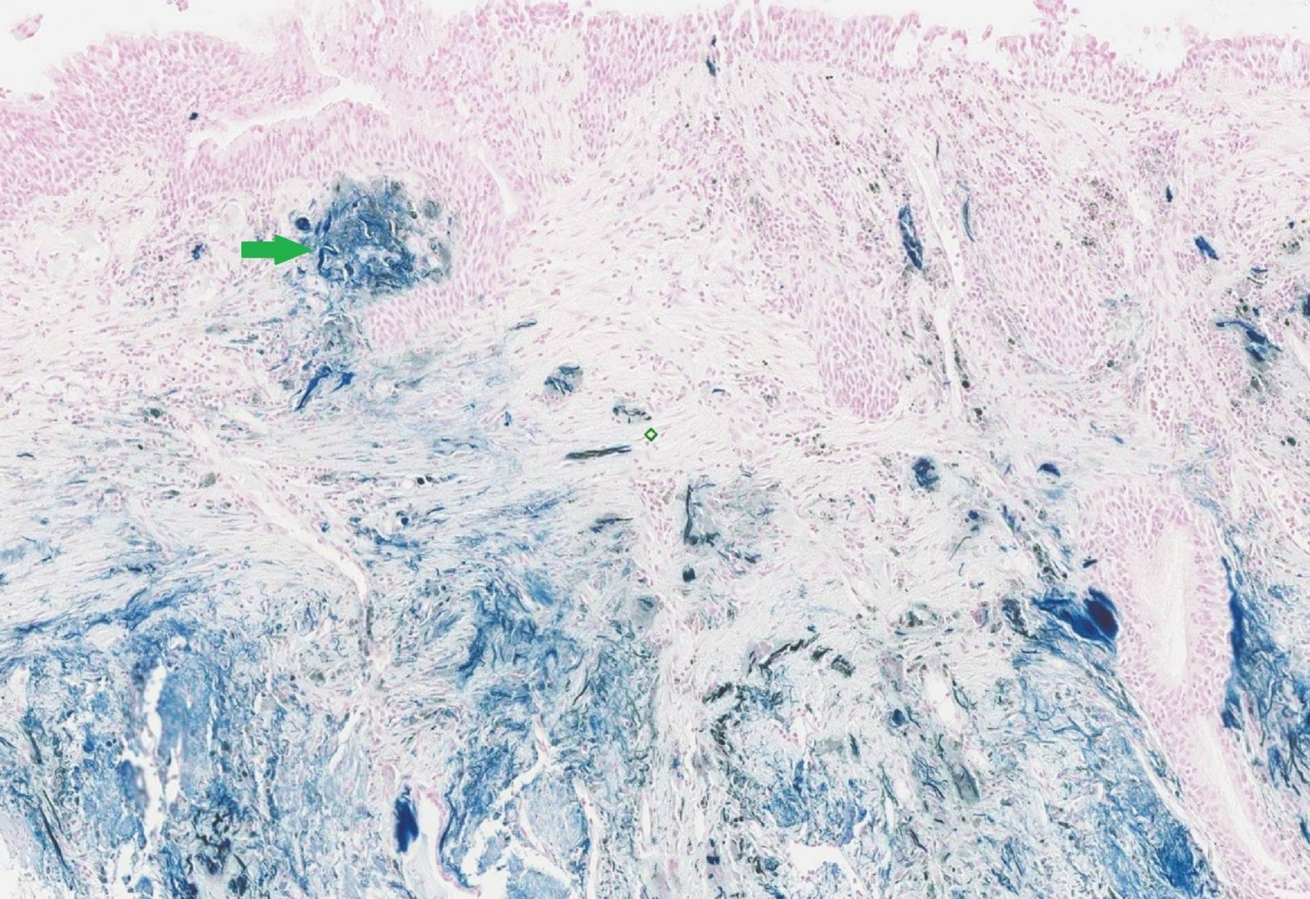
Prussian blue stain of the bronchial biopsy showing evidence of iron deposition (green arrow)

There was blackish pigmentation noted in the bronchial mucosa secondary to the iron deposition given her history of aspirating the iron pill. The pathology of the bronchial mucosa was reported as “ulceration and necrosis of bronchial wall, with acute inflammation, fibrinous exudate and prominent stromal iron deposition” (Figures 3-5).

**Figure 6:**
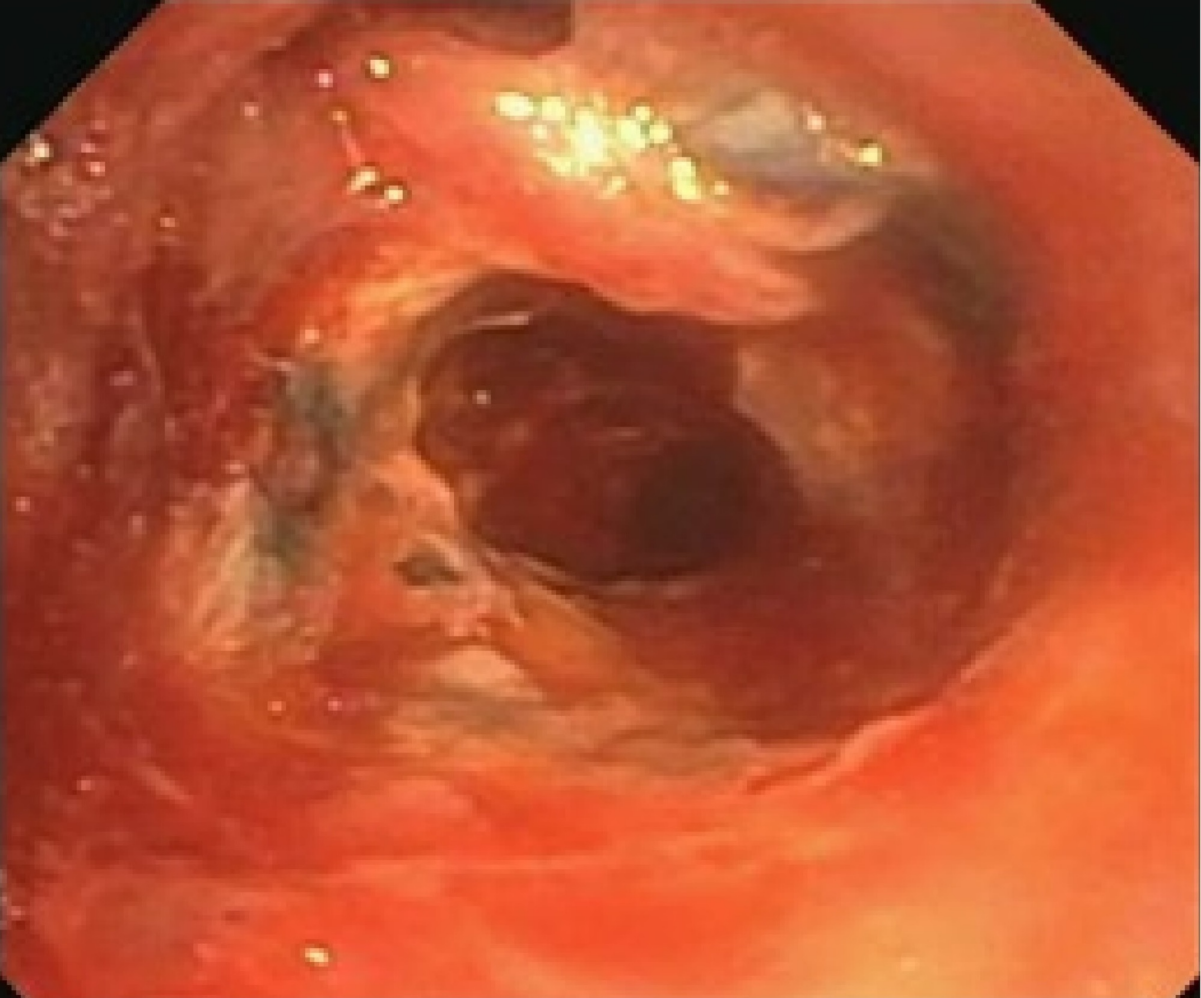
Bronchus intermedius after cryotherapy and debridement

**Figure 7:**
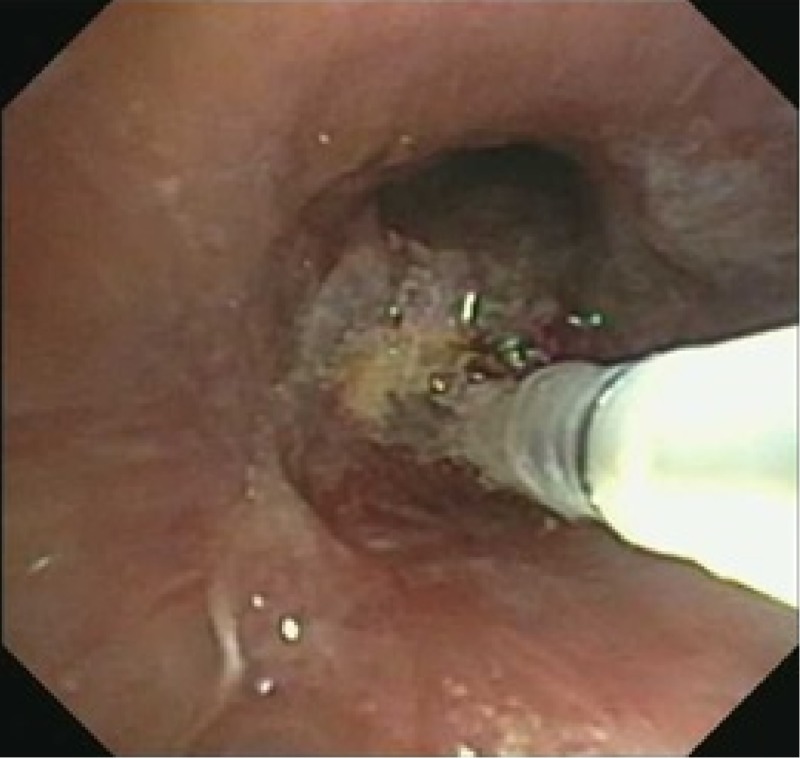
Balloon dilation of the right lower lobe

**Figure 8:**
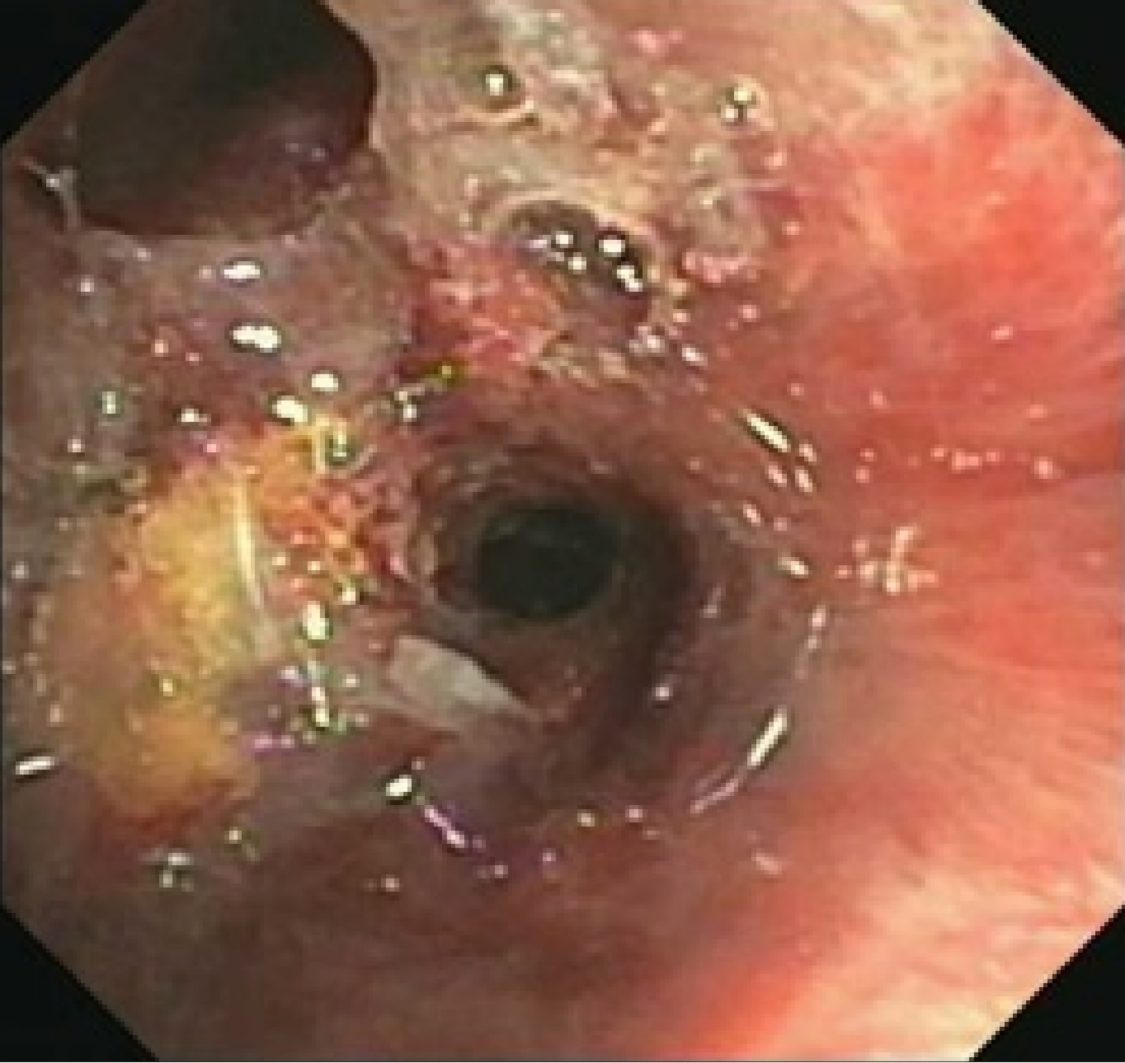
Right lower lobe after balloon dilation

**Figure 9:**
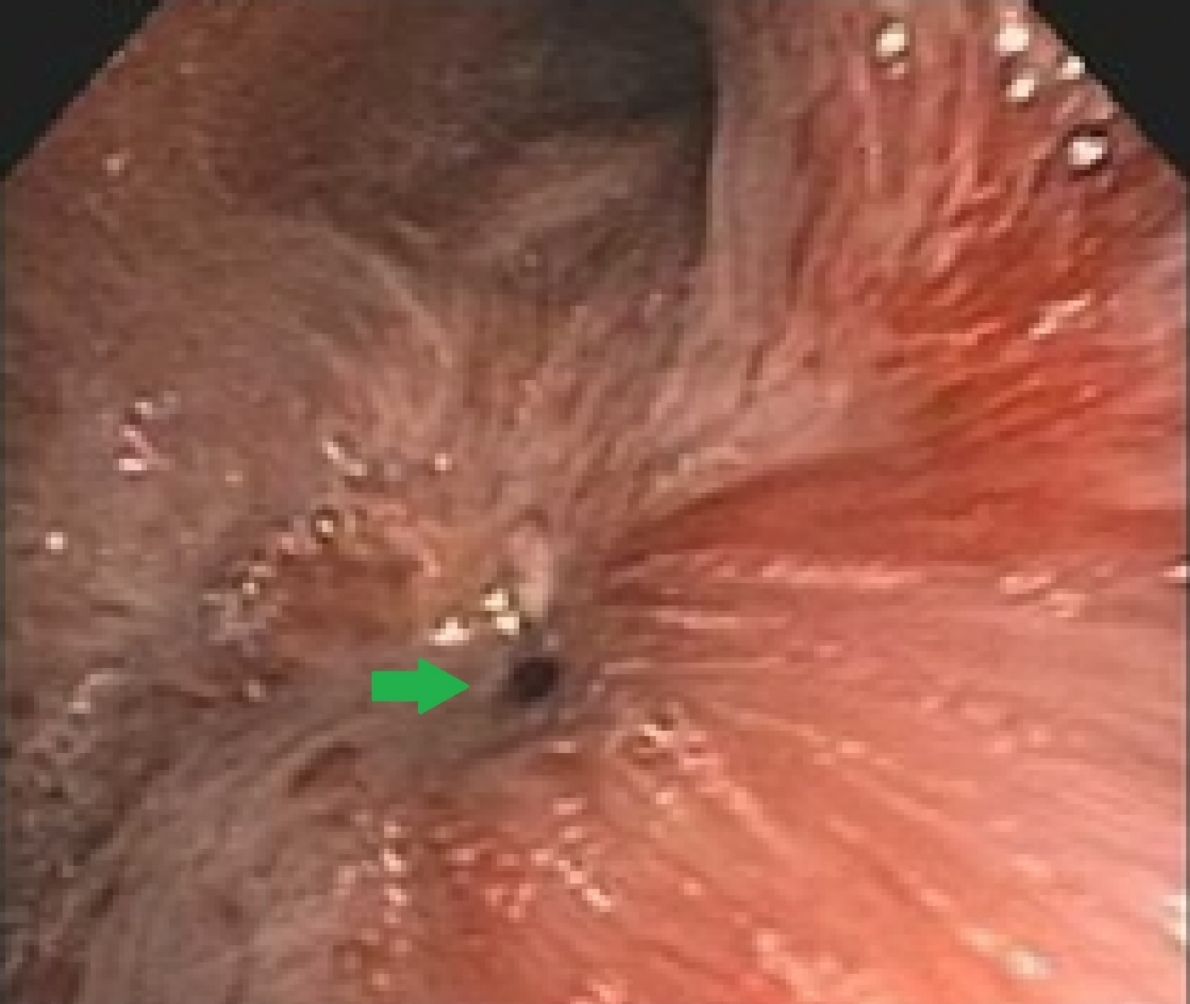
Stenosis of the right lower lobe (green arrow) several months after balloon dilation

A rigid bronchoscopy was performed for cryo-debridement of necrotic tissues and with mitomycin application to the lesion. She had two follow-up bronchoscopies done four weeks apart which showed worsening fibrosis and stenosis of the RBI. Bronchoscopic balloon dilation was attempted with minimal improvement (Figures 6-9).

## Discussion

Most cases of foreign body aspiration occur in children; however, approximately 20% of foreign body aspiration occurs in adults [3]. Patient populations at the highest risk of foreign body aspiration include the elderly, especially in those who have primary neurologic disorders, decreased gag reflexes due to alcohol, seizures, strokes, trauma, dementia, and Parkinsonism [4].

The diagnosis of foreign body aspiration is difficult, as physical symptoms are non-specific and chest radiographs can be normal in about 25% of cases [5]. Coughing, wheezing, hemoptysis, dyspnea, and decreased air entry can be the characteristic of one aspirating a foreign body [3,5]. Foreign body aspirations are often misdiagnosed as asthma or chronic pneumonia [2]. The CT findings can include unilateral lung hyper-lucency, bronchiectasis, atelectasis, lobar consolidation or pleural effusion [3]. Specifically, IPAS has been reported to be seen on CT scan as circumferential thickening of the bronchus intermedius [1].

Iron sulfate is unlike other pills in that it disintegrates in the airway, hence the Perls' Prussian blue iron stain can determine the presence of an aspirated iron tablet. Iron sulfate causes caustic necrosis by local production of cytotoxic oxidants and free radicals. It can cause bronchovascular necrosis leading to severe and fatal hemorrhage and late complications include bronchial necrosis and stenosis [6].

In our case, our patient had unfortunately aspirated an iron tablet causing local irritation, inflammation, and bronchial stenosis of the RBI. Despite repeated bronchoscopies, cryo-therapy, and balloon dilation, there remained a stenosis of the bronchial airway causing her symptoms of persistent dyspnea and wheezing. Physicians should be very prudent with prescribing iron sulfate or any other pills in individuals who are at high risk of aspiration. With long-standing organic foreign bodies that cause the formation of granulation tissue, several case series have reported that systemic glucocorticoids 12 to 24 hours before removal may aid in extraction by reducing inflammation [3]. If there is an aspiration event, prompt diagnosis and rigid bronchoscopy remain the gold standard for treatment as it offers superior airway control, suction, and extraction capabilities.

## Conclusions

Obliterating bronchiolitis is common in the aspiration of organic foreign bodies such as IPAS. Prompt diagnosis and timely management should be initiated to prevent further worsening of the disability.
